# The BBM2‐BZR4‐*GrxC2.2* Module Regulates Rice Embryogenesis Independently of the BR Pathway

**DOI:** 10.1111/pbi.70408

**Published:** 2025-11-01

**Authors:** Jia‐Wen Yu, Jin‐Dong Wang, Ying‐Mei Deng, Li‐Jun Kan, Cheng‐Chao Zhu, Meng‐Fan Jiang, Dong‐Sheng Zhao, Xiao‐Lei Fan, Chang‐Quan Zhang, Li‐Chun Huang, Qiao‐Quan Liu, Qian‐Feng Li

**Affiliations:** ^1^ Jiangsu Key Laboratory of Crop Genomics and Molecular Breeding/Zhongshan Biological Breeding Laboratory/Key Laboratory of Plant Functional Genomics of the Ministry of Education, College of Agriculture Yangzhou University Yangzhou China; ^2^ Co‐Innovation Center for Modern Production Technology of Grain Crops of Jiangsu Province/Key Laboratory of Crop Genetics and Physiology of Jiangsu Province Yangzhou University Yangzhou China

**Keywords:** *BBM2*, *BZR4*, embryoless seeds, milled rice rate, rice

Brassinosteroids (BRs) are sterol‐derived phytohormones that play a crucial role in regulating various agronomic traits related to crop yield (Song et al. 2023; Zhang, Meng, et al. 2024). It is therefore believed that precise manipulation of the BR pathway can effectively improve both crop yield and quality traits, thereby making a substantial contribution to the next green revolution (Yang et al. 2024; Li et al. 2023). BZR‐family transcription factors are pivotal positive regulators within the BR signalling cascade. In rice, knocking out *OsBZR1* results in a pleiotropic phenotype characterised by reduced grain size, compact plant architecture and increased resistance to preharvest sprouting (PHS) (Xiong et al. 2022). However, a recent study reported that BZR5 functions as a negative regulator of BR signalling and rice grain size, revealing an unconventional role for BZRs in rice (Zhang, Wu, et al. 2024). This implies that functional redundancy, diversification and even antagonism coexist among different BZR members. Therefore, elucidating the biological functions of distinct BZR members will improve our understanding of the BR‐governed genetic network and advance the molecular breeding practices.

In this study, we generated loss‐of‐function mutants of all *BZRs*. Interestingly, only the *bzr4* mutant exhibited an embryoless phenotype (Figure S1). We therefore classified the seeds of the *bzr4* mutants into three groups: normal embryo, abnormal embryo, and no embryo (Figure 1a). Quantitative data showed that around 80% of *bzr4* seeds were embryoless (Figure 1b). To determine whether *BZR4* controls embryogenesis via the BR pathway, we examined the seeds of the other BR‐deficient and BR‐insensitive mutants. However, no aberrant embryoless phenotypes were observed (Figure S2). Although the in vitro and in vivo experiments confirmed that BZR4 directly interacts with BZR1 and GSK2 (Figures S3 and S4), *BZR1* overexpression or *GSK2* mutation could not restore the embryoless phenotype of the *bzr4* mutant (Figure 1c,d). Overall, genetic evidence demonstrated that *BZR4* regulates rice embryogenesis independently of BR signalling.

**FIGURE 1:**
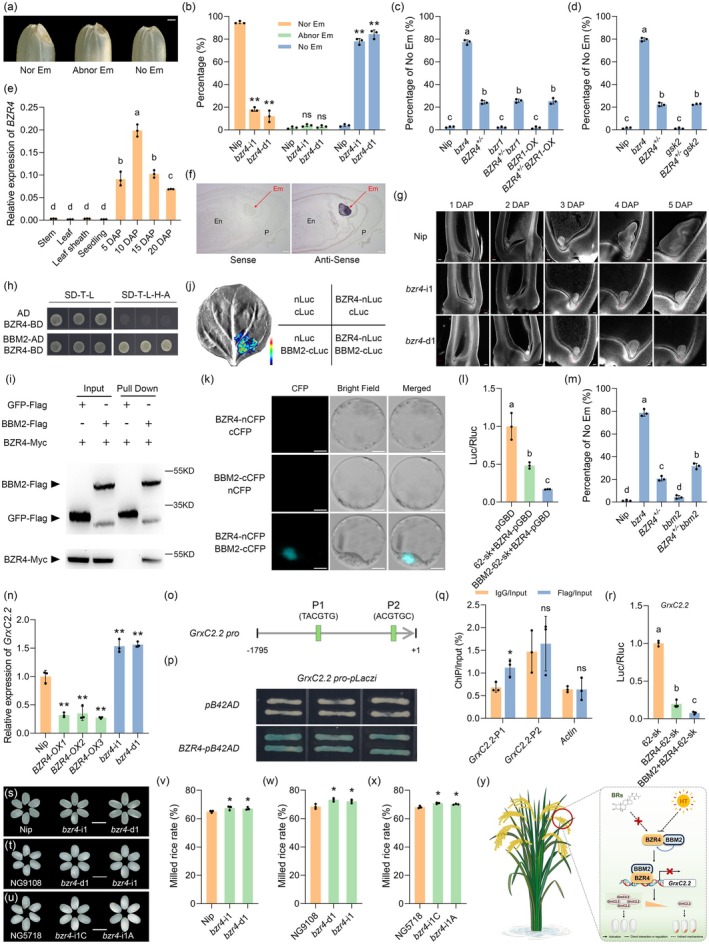
BBM2‐BZR4‐*GrxC2.2* module is involved in regulating rice embryogenesis. (a) Seed embryonic morphology. Nor Em, normal embryo; Abnor Em, abnormal embryo; No Em, no embryo. Scale bar, 5 mm. (b) The percentage of different types of embryo. Percentage of embryoless seeds in *bzr4*, *bzr1* and *BZR1‐OX* single and double mutants (c), and *gsk2* single and double mutants (d). (e) Spatiotemporal expression patterns of *BZR4*. (f) In situ hybridisation of *BZR4* expression in seeds at 4 DAP. Em, embryo; En, endosperm; P, pericarp. Scale bar, 100 μm. (g) Embryonic morphology of Nip and *bzr4* at different developmental stages, as observed using laser‐scanning confocal microscopy. Scale bar, 50 μm. Yeast two‐hybrid analysis (h), pull‐down experiment (i), split‐luciferase complementation assay (j), and bimolecular fluorescence complementation analysis (k) to verify the interaction between BBM2 and BZR4. In (i), anti‐Myc and anti‐Flag antibodies were used to detect BBM2‐Flag and BZR4‐Myc, respectively. (l) BBM2 promotes the transcriptional suppression activity of BZR4. (m) The percentage of embryoless seeds in *bzr4* and *bbm2* single and double mutants. (n) *GrxC2.2* expression in *BZR4* overexpression lines and *bzr4* mutants. (o) A schematic representation of the E‐box in the *GrxC2.2* promoter. Yeast one‐hybrid assay (p) and ChIP‐qPCR analysis (q) for analysing the interaction between BZR4 and the *GrxC2.2* promoter. (r) Dual‐luciferase reporter analysis of BZR4 and BBM2 on *GrxC2.2* expression. Phenotypes (s–u) and quantitative data (v–x) of the milled rice in *bzr4* mutants with the genetic backgrounds of Nipponbare, NG9108 and NG5718, respectively. Scale bar, 5 mm. (y) A model of the BBM2‐BZR4‐*GrxC2.2* regulatory cascade that regulates rice embryogenesis. In b–e, l–n, q, r, and v–x, all data are means ± SD (*n* = 3). In b, n, q and v–x, *p < 0.05; **p < 0.01; ns, no significance (Student's *t*‐test). In c–e, l, m and r, different letters indicate significant differences (*p* < 0.05, one‐way ANOVA with two‐sided Tukey's HSD test).

Several experiments were conducted to shed light on how *BZR4* controls embryogenesis. RT‐qPCR results showed that *BZR4* is predominantly expressed in seeds (Figure 1e). An in situ hybridisation experiment further demonstrated its embryo‐specific expression (Figure 1f). Furthermore, the BZR4 protein was localised in both the cytoplasm and the nucleus, exhibiting strong transcriptional repressor activity (Figure S5). The anthers, pistils and ovaries of *bzr4* mutants appeared similar to those of the wild type (Figure S6), confirming that maternal floral organs develop normally. Confocal laser‐scanning microscopy of developing seeds revealed that *bzr4* mutants experienced arrest of embryogenesis between 3 and 4 days after pollination (DAP) (Figure 1g). Further corroboration came from paraffin section analysis, which showed that most *bzr4* seeds lacked an embryonic structure by 15 DAP (Figure S7). These data demonstrate that the developmental lesion in the *bzr4* mutants occurs at the globular stage of embryogenesis. Additionally, a potential BZR4‐interacting protein, BBM2 (a close homologue of BBM1; Khanday et al. 2019), was identified by screening a yeast two‐hybrid library. RT‐qPCR results showed that *BBM2* is also predominantly expressed in seeds (Figure S8). Further experiments demonstrated that BZR4 interacts with BBM2 in vitro and in vivo (Figure 1h–k). A dual‐luciferase reporter assay revealed that BBM2 significantly enhances the transcriptional repressor activity of BZR4 (Figure 1l). Although mutation of *BBM2* alone had no effect on rice embryogenesis (Figure S9), mutation of this gene significantly increased the proportion of embryoless seeds in the *bzr4* mutant (Figure 1m).

To further clarify the downstream targets of BZR4 in orchestrating embryogenesis, the candidate gene *GrxC2.2* was investigated, given that its overexpression also results in embryoless seeds in rice (Liu et al. 2019). Notably, *GrxC2.2* expression was repressed in *BZR4*‐overexpression lines and promoted in *bzr4* mutants (Figures 1n and S10), indicating that BZR4 negatively regulates *GrxC2.2* expression. Promoter analysis revealed the presence of two E‐boxes in the *GrxC2.2* promoter (Figure 1o). Consequent yeast one‐hybrid and ChIP‐qPCR assays confirmed that BZR4 can directly bind to the *GrxC2.2* promoter (Figure 1p,q). A further dual‐luciferase reporter assay confirmed that BZR4 significantly suppresses *GrxC2.2* expression alone or in conjunction with BBM2 (Figure 1r). Consistently, knocking out *BBM2* did not affect *GrxC2.2* gene expression, but it can further promote *GrxC2.2* expression in the *bzr4* mutant (Figure S11).

The *BZR4* mutation in Nipponbare significantly increased the milled rice rate (Figure 1s,v). To confirm this effect, two popular commercial rice varieties from Jiangsu (NG9108 and NG5718) were subjected to gene editing and subsequent evaluation (Figures S12 and S13). The phenotyping results indicated that the *BZR4* mutation in both varieties resulted in more than 85% embryoless seeds and a significantly higher milled rice rate (Figure 1t,u,w,x). Therefore, the *BZR4* mutation indeed enhances rice processing quality and has industrial potential. Recently, Wang et al. (2025) demonstrated that BZR4 modulates rice embryogenesis in a temperature‐dependent manner. This provides a new approach to designing temperature‐regulated embryoless germplasm. However, our research provides robust genetic evidence demonstrating that BZR4 regulates rice embryogenesis independently of the BR pathway. We have also successfully established a BBM2‐BZR4‐*GrxC2.2* molecular module to illustrate this regulation. In summary, the establishment of the BR‐independent BBM2–BZR4–*GrxC2.2* regulatory module in this study, alongside the work of Wang et al. (2025), has significantly advanced our understanding of the mechanisms that orchestrate rice embryogenesis (Figure 1y).

## Author Contributions

Q.‐F.L. and Q.‐Q.L. conceived the project and supervised the study. J.‐W.Y., J.‐D.W., Y.‐M.D., L.‐C.H., L.‐J.K., C.‐C.Z., M.‐F.J., D.‐S.Z., X.‐L.F. and C.‐Q.Z. conducted the experiments. Q.‐F.L., Q.‐Q.L. and L.‐C.H. analysed the data. J.‐W.Y. and L.‐C.H. wrote the manuscript. Q.‐F.L. and Q.‐Q.L. revised the manuscript. All authors read and approved the manuscript.

## Conflicts of Interest

The authors declare no conflicts of interest.

## Supporting information


**Figures S1–S13. Table S1**.

## Data Availability

The authors have nothing to report.
