# Supplementary material for: The BBM2‐BZR4‐GrxC2.2 Module Regulates Rice Embryogenesis Independently of the BR Pathway
Source: Plant Biotechnol J. 2025 Nov 1;24(3):1649–51. doi: 10.1111/pbi.70408 (PMC12946489; doi:10.1111/pbi.70408)
Supplement: Supplementary file 1 — Figures S1–S13. Table S1. [file PBI-24-1649-s001.docx]

**Supporting information**

**The BBM2-BZR4-*GrxC2.2* module regulates rice embryogenesis independently of the BR pathway**

Jia-Wen Yu^1,†^, Jin-Dong Wang^1,2,†^, Ying-Mei Deng^1,†^, Li-Jun Kan^1^, Cheng-Chao Zhu^1^, Meng-Fan Jiang^1^, Dong-Sheng Zhao^1,2^, Xiao-Lei Fan^1,2^, Chang-Quan Zhang^1,2^, Li-Chun Huang^1,2,*^, Qiao-Quan Liu^1,2,*^ and Qian-Feng Li^1,2,*^

**This file includes:**

**Figures S1 to S13**

**Table S1**

**Materials and Methods**

**SI References**

**
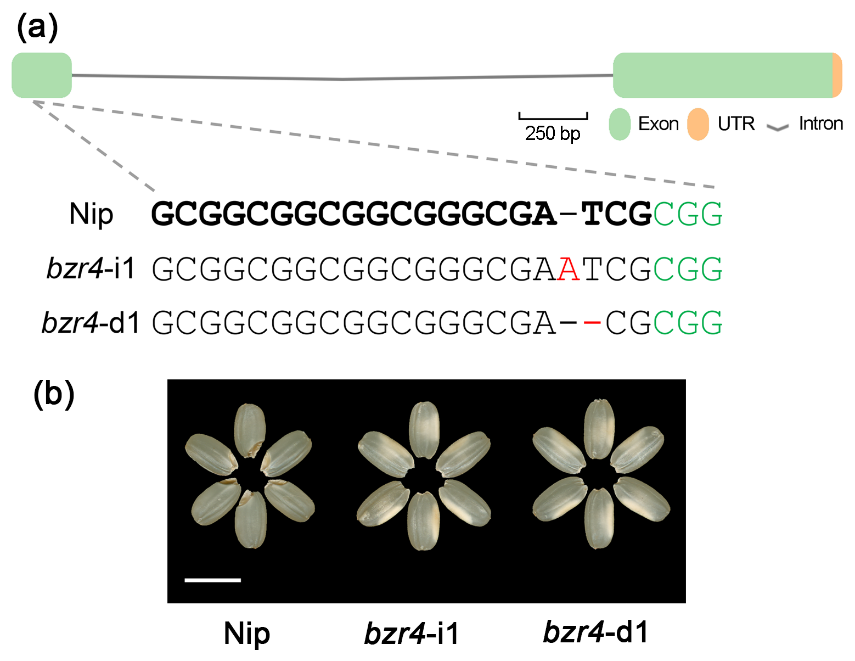
**

**Figure S1** Phenotypes of brown rice in the *bzr4* mutant in the Nip background. (a) Gene editing target in the *BZR4* gene and the corresponding mutation information for the *bzr4* mutants. (b) Phenotypes of *bzr4* brown rice. Scale bar, 5 mm.

**
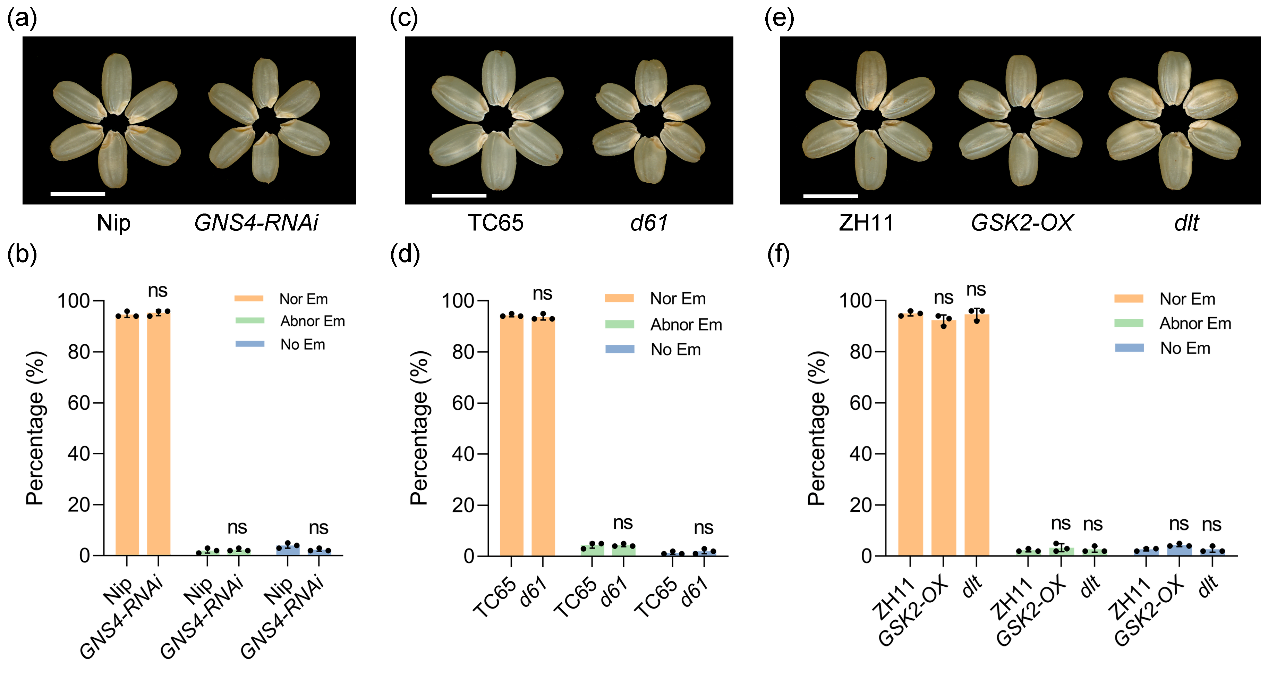
**

**Figure S2** Phenotypes of brown rice of BR deficient and insensitive genetic materials. Phenotype of brown rice (a) and percentage of different types of embryos in *GNS4-RNAi* rice and the wild-type Nip. Phenotype of brown rice (c) and percentage of different types of embryos in *d61* mutant and the wild-type TC65. (e, f) Embryo phenotype and percentage of different types of embryos in *GSK2-OX* and *dlt* materials. Scale bar, 5 mm. All data are means ± SD (n=3). *P<0.05; **P<0.01; ns, no significance (Student’s *t*-test).

**
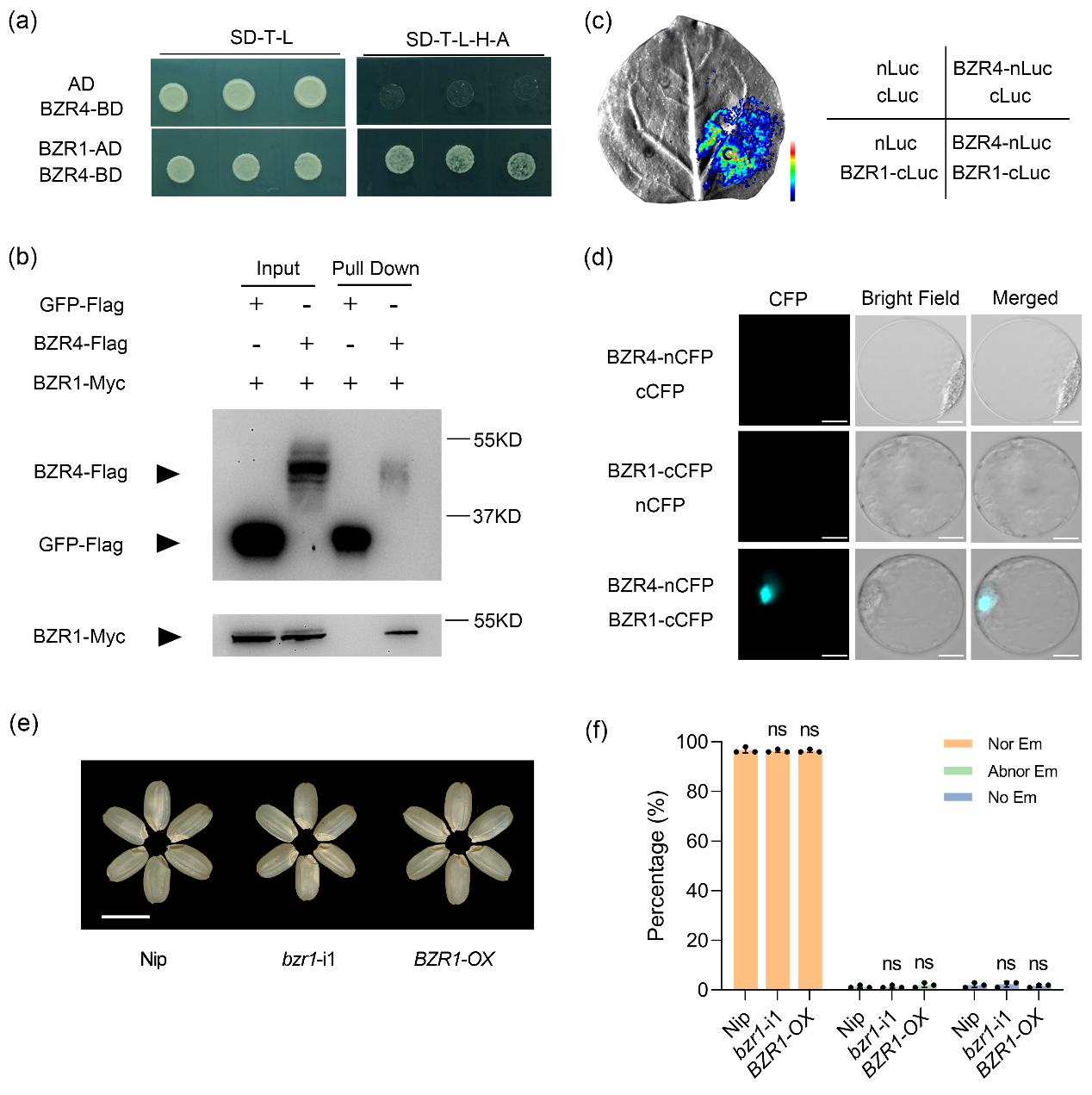
**

**Figure S3** Interaction analysis of the BZR1 and BZR4 proteins, and analysis of the embryo phenotypes of the *BZR1*-related rice materials. The protein-protein interaction between the BZR1 and BZR4 proteins was analyzed using yeast two-hybrid analysis (a), pull-down experiments (b), split-luciferase complementation assays (c) and bimolecular fluorescence complementation (BiFC) analysis (d). In (b), anti-Myc and anti-Flag antibodies were used to detect BZR1-Myc and BZR4-Flag, respectively. The plus (+) and minus (-) signs indicate the presence or absence of the indicated protein in each group. In (c), the experiment was performed in the leaf epidermal cells of *Nicotiana benthamiana*, using three construct combinations as negative controls: *nLUC* and *cLUC*; *BZR1-cLUC* and *nLUC*; and *cLUC* and *BZR4-nLUC*. In (d), rice protoplasts were used for transformation, with two sets of plasmid groups being used as negative controls: *BZR1-cCFP* and *nCFP*, and *cCFP* and *BZR4-nCFP*. Scale bar, 5 μm. Embryo phenotypes (e) and the percentage of different types of embryos (f) in the seeds of Nip and *BZR1*-related rice materials. In (e), the scale bar is 5 mm. In (f), all data are means ± SD (n = 3) and ns indicates no significance (Student’s *t*-test).

**
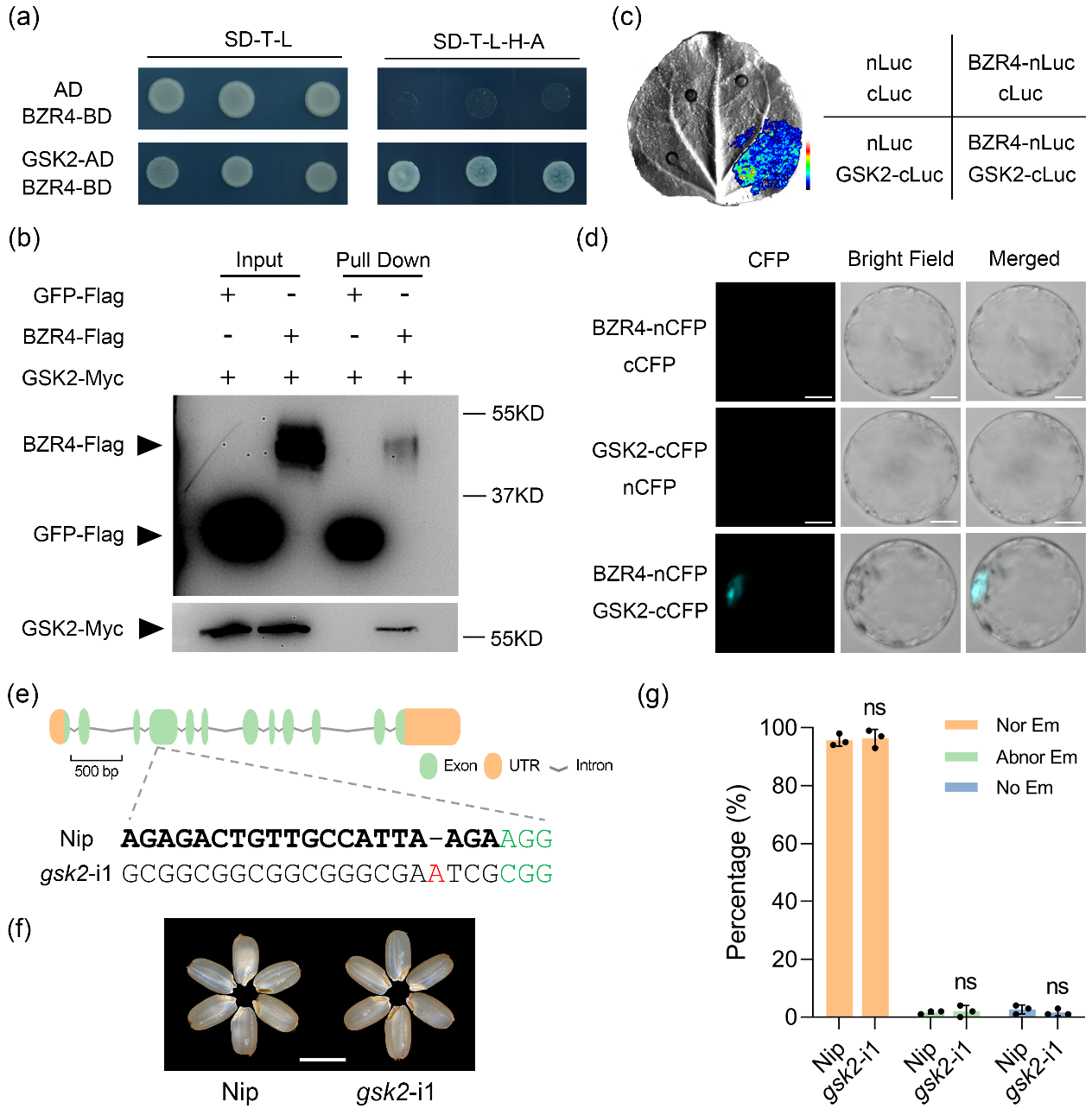
**

**Figure S4** Interaction analysis of the GSK2 and BZR4 proteins, and analysis of the embryo phenotypes of the *gsk2* mutant. The protein-protein interaction between the BZR1 and GSK2 proteins was analyzed using yeast two-hybrid analysis (a), pull-down experiments (b), split-luciferase complementation assays (c) and BiFC analysis (d). In (b), anti-Myc and anti-Flag antibodies were used to detect GSK2-Myc and BZR4-Flag, respectively. The plus (+) and minus (-) signs indicate the presence or absence of the indicated protein in each group. In (c), the experiment was performed in the leaf epidermal cells of *Nicotiana benthamiana*, using three construct combinations as negative controls: *nLUC* and *cLUC*; *GSK2-cLUC* and *nLUC*; and *cLUC* and *BZR4-nLUC*. In (d), two sets of plasmid groups were used as negative controls: *GSK2-cCFP* and *nCFP*, and *cCFP* and *BZR4-nCFP*. Scale bar, 5 μm. (e) Gene editing target in the *GSK2* gene and the corresponding mutation information for the *gsk2* mutant. (f) Embryo phenotypes of the *gsk2* mutant. Scale bar, 5 mm. (g) The percentage of different embryo types in Nip and *gsk2* mutant seeds. All data are means ± SD (n = 3). ns, no significance (Student’s *t*-test).

**
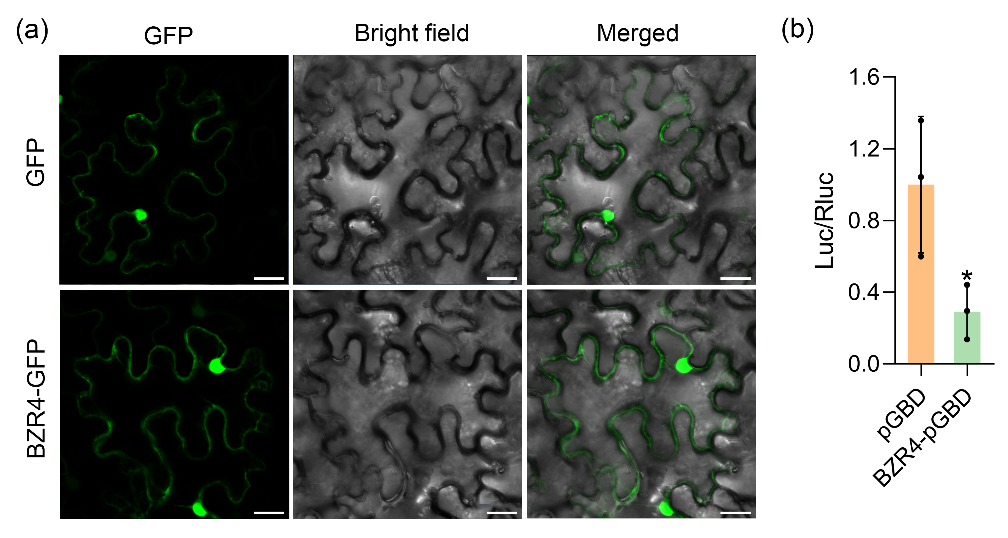
**

**Figure S5** Analysis of the subcellular localization and transcription activation activity of BZR4. (a) The subcellular localization of BZR4 in the leaf epidermal cells of *Nicotiana benthamiana*. Scale bar, 20 μm. (b) Transcription activation activity of BZR4. All data are means ± SD (n = 3). *P < 0.05 (Student’s *t*-test).

**
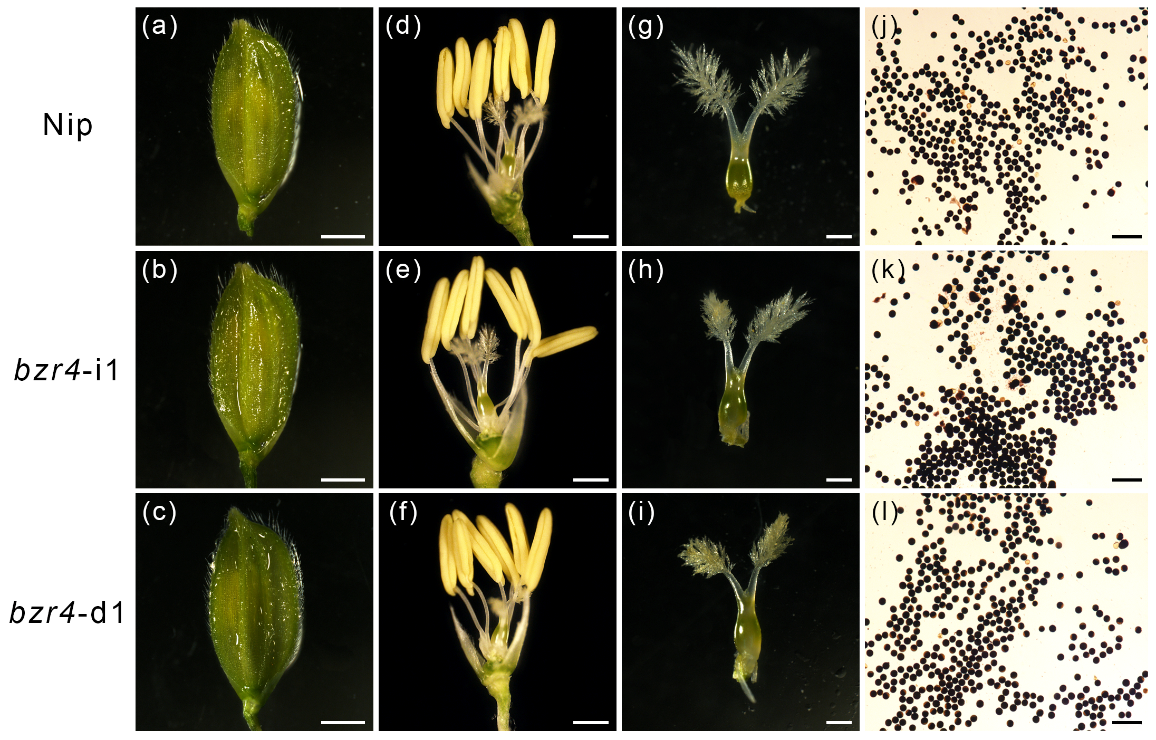
**

**Figure S6** Analysis of the glumes, anthers, ovaries and pollen fertility in Nip and *bzr4* mutants. (a-c) Glume morphology in Nip and *bzr4* mutants. Scale bar, 5 mm. (d-f) The morphology of the anthers of Nip and the *bzr4* mutants. Scale bar, 500 μm. (g-i) The morphology of the ovaries of Nip and the *bzr4* mutants. Scale bar, 200 μm. (j-l) Pollen fertility of Nip and the *bzr4* mutants. Scale bar, 100 μm.

**
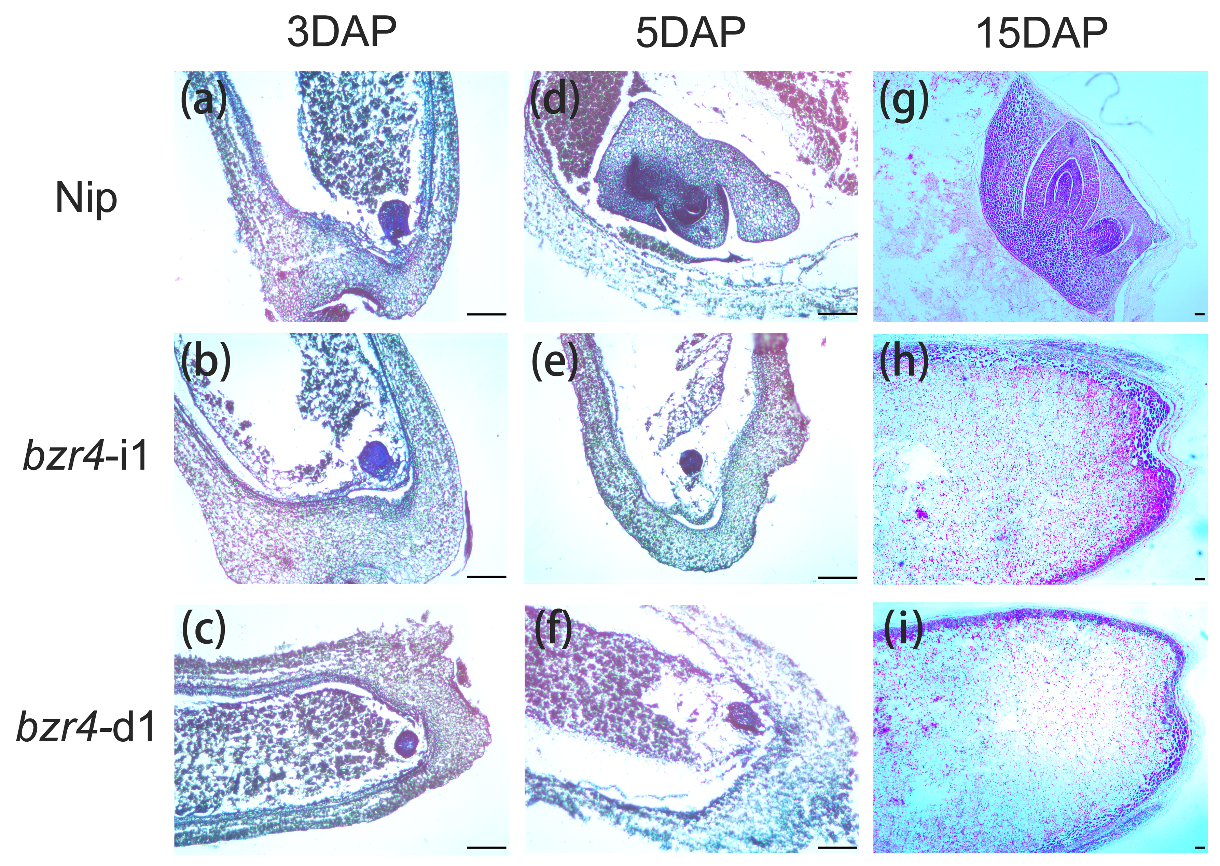
**

**Figure S7** Median longitudinal sections of Nip and *bzr4* embryos at various developmental stages. Developing Nip, *bzr4*-i1, and *bzr4*-d1 embryos at 3, 5 and 15 days after pollination (DAP), respectively. Scale bar, 200 μm in (a-f) and 50 μm in (g-i).

**
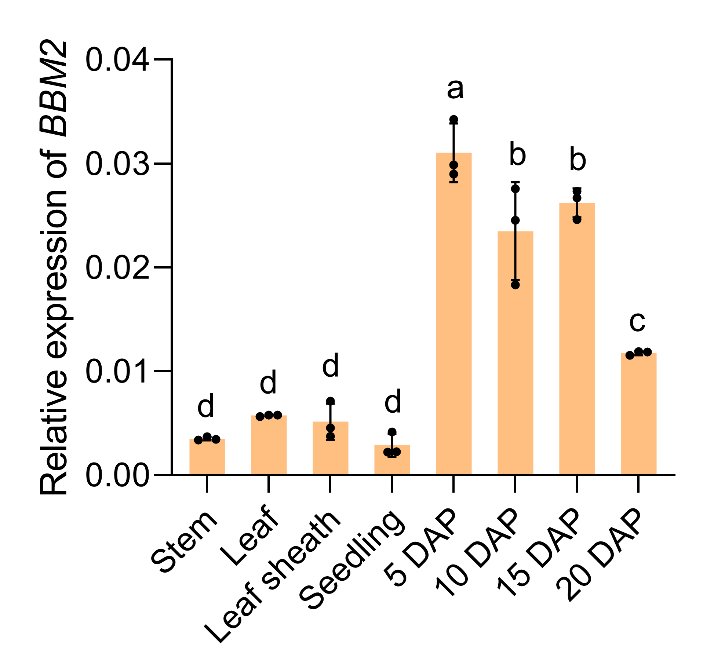
**

**Figure S8** Spatiotemporal expression patterns of *BBM2*. *OsActin01* was used as an internal control for normalization. All data are means±SD (n = 3). Different letters indicate significant differences (P<0.05, one-way ANOVA with two-sides Turkey’s HSD test).

**
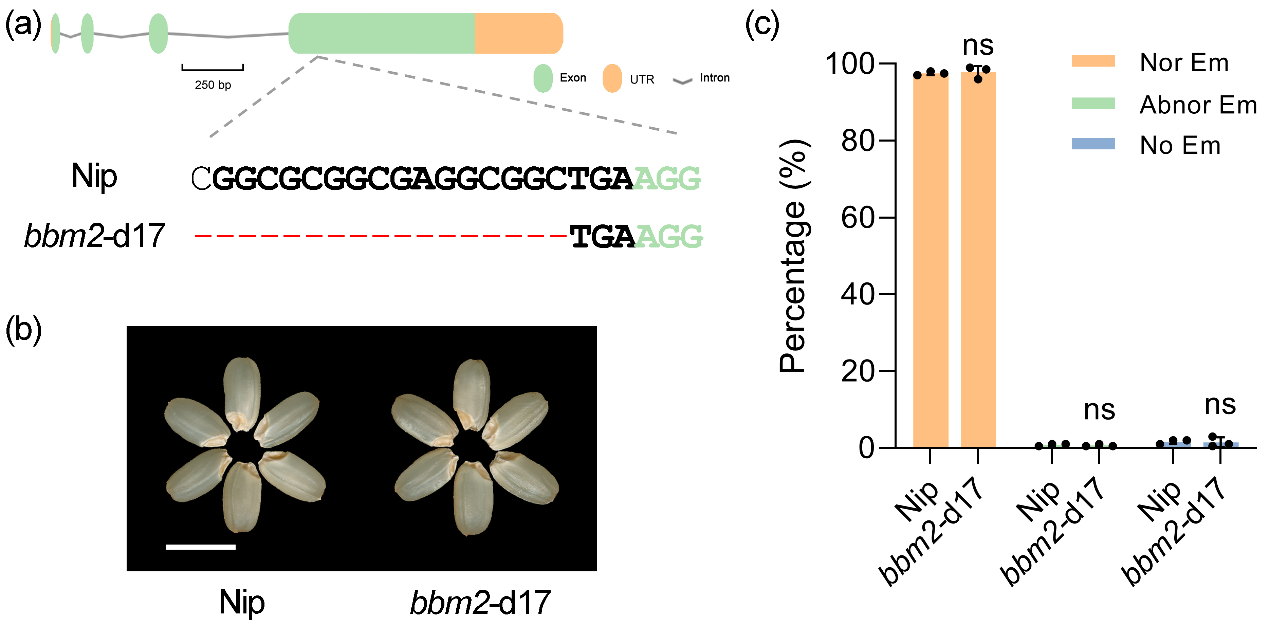
**

**Figure S9** Analysis of *bbm2* mutant seeds. (a) Gene editing target in the *BBM2* gene and the corresponding mutation information for the *bbm2* mutants. (b) Embryo phenotypes of the *bbm2* mutant. Scale bar, 5 mm. (c) The percentage of different embryo types in Nip and *bbm2* mutant seeds. All data are means ± SD (n = 3). ns, no significance (Student’s *t*-test).

**
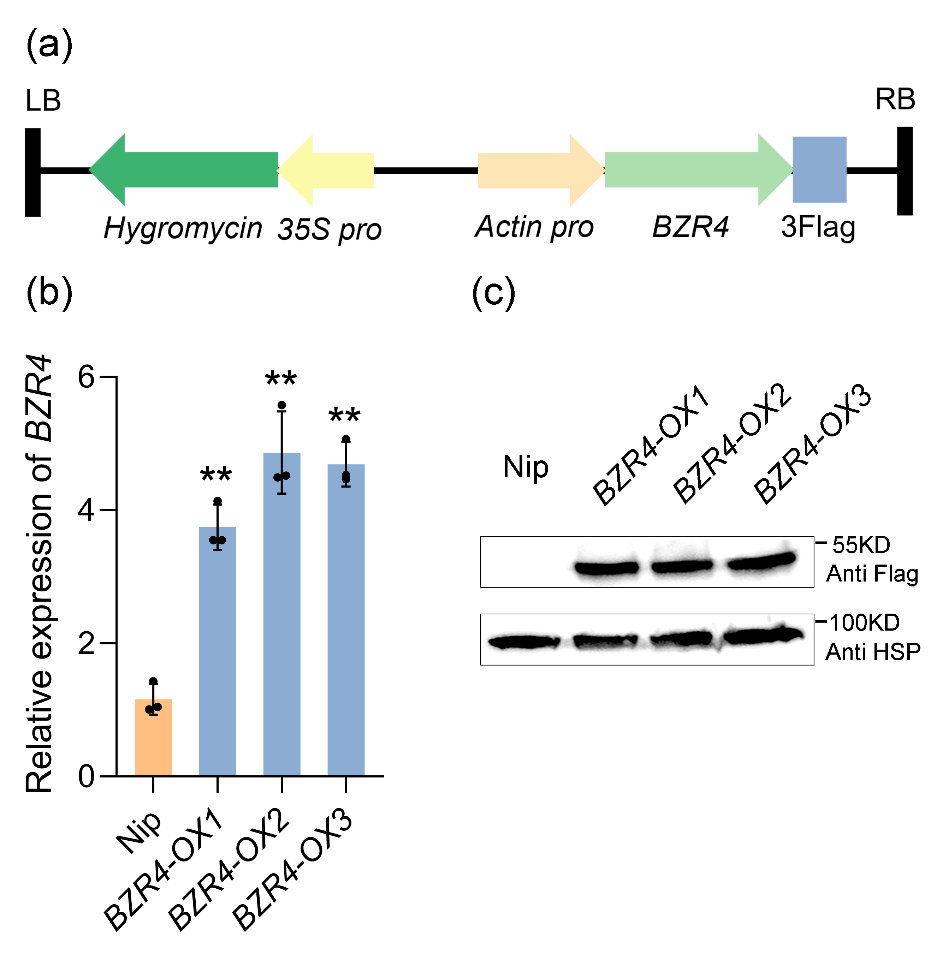
**

**Figure S10** Generation of the *BZR4* overexpression transgenic rice. (a) Structural diagram of the T-DNA region of the *BZR4* overexpression vector. LB, left border; RB, right border. (b) Analysis of *BZR4* expression in *BZR4*-overexpressing rice. All data are means ± SD (n = 3). **P < 0.01 (Student’s *t*-test). (c) Analysis of BZR4 protein abundance in the *BZR4* overexpression lines. BZR4 and HSP protein amounts were detected using anti-Flag and anti-HSP antibodies, respectively.

**
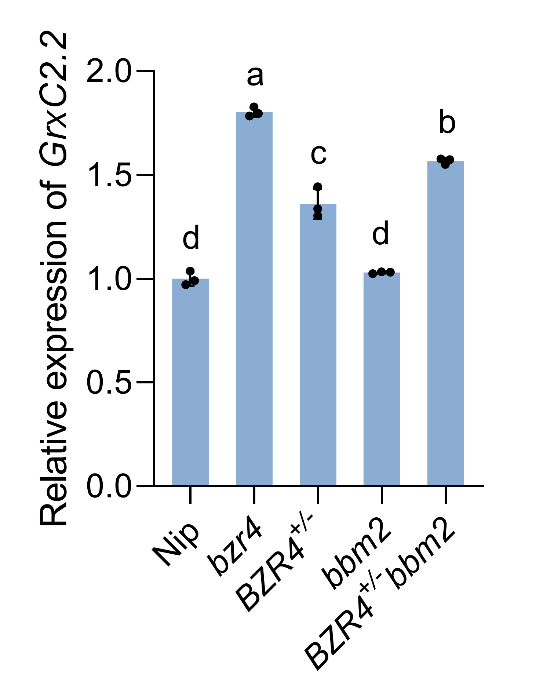
**

**Figure S11** Relative expression of *GrxC2.2* in *bzr4* and *bbm2* single and double mutants. *OsActin01* was used as an internal control for normalization. All data are means±SD (n = 3). Different letters indicate significant differences (P<0.05, one-way ANOVA with two-sides Turkey’s HSD test).

**
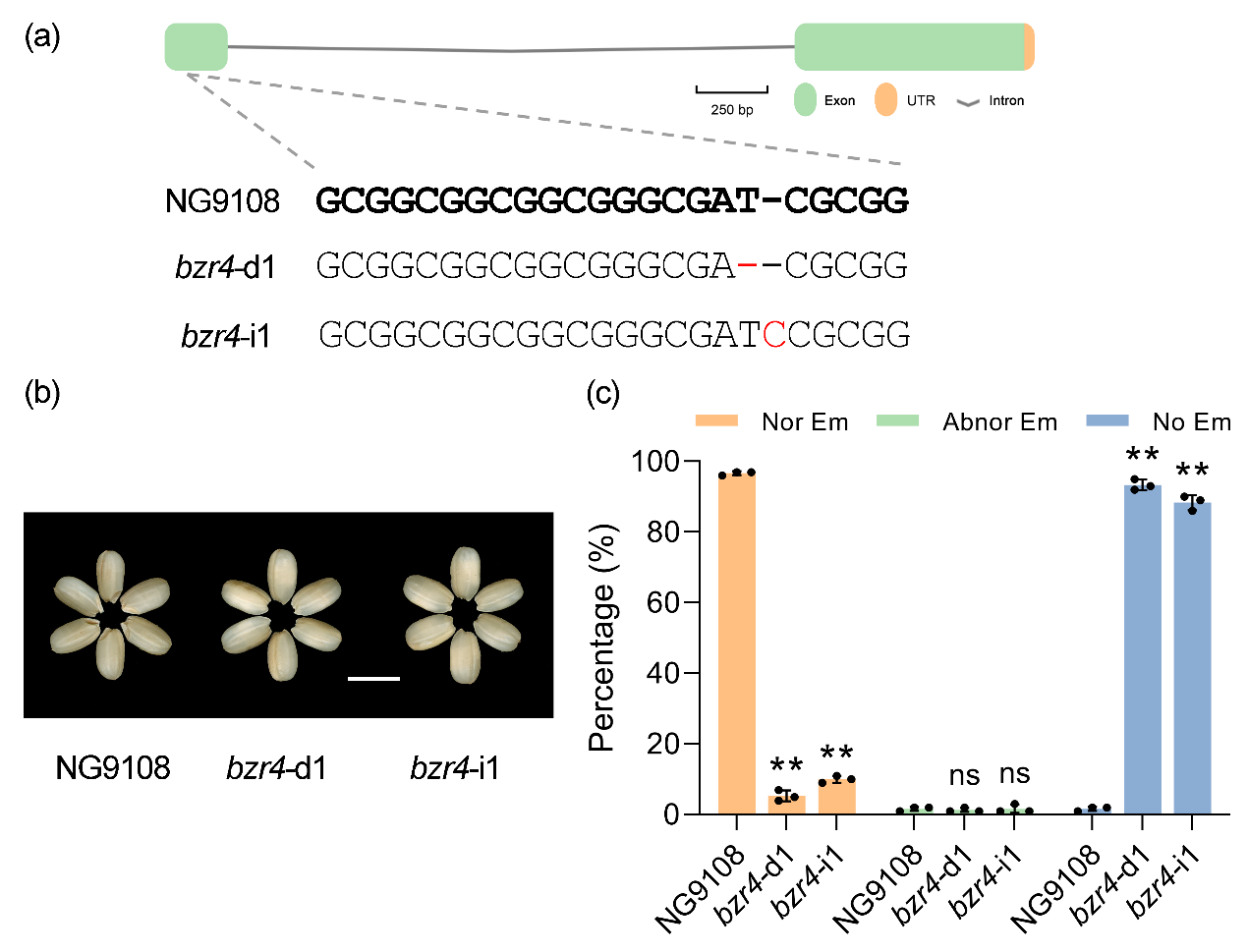
**

**Figure S12** Analysis of *bzr4* mutants in the NG9108 background. (a) Gene editing target in the *BZR4* gene and the corresponding mutation information for the *bzr4* mutants. (b) Embryo phenotypes of *bzr4* mutants. Scale bar, 5 mm. (c) The percentage of different embryo types in the seeds of NG9108 and *bzr4* mutants. All data are means ± SD (n = 3). **P < 0.01; ns, no significance (Student’s *t*-test).

**
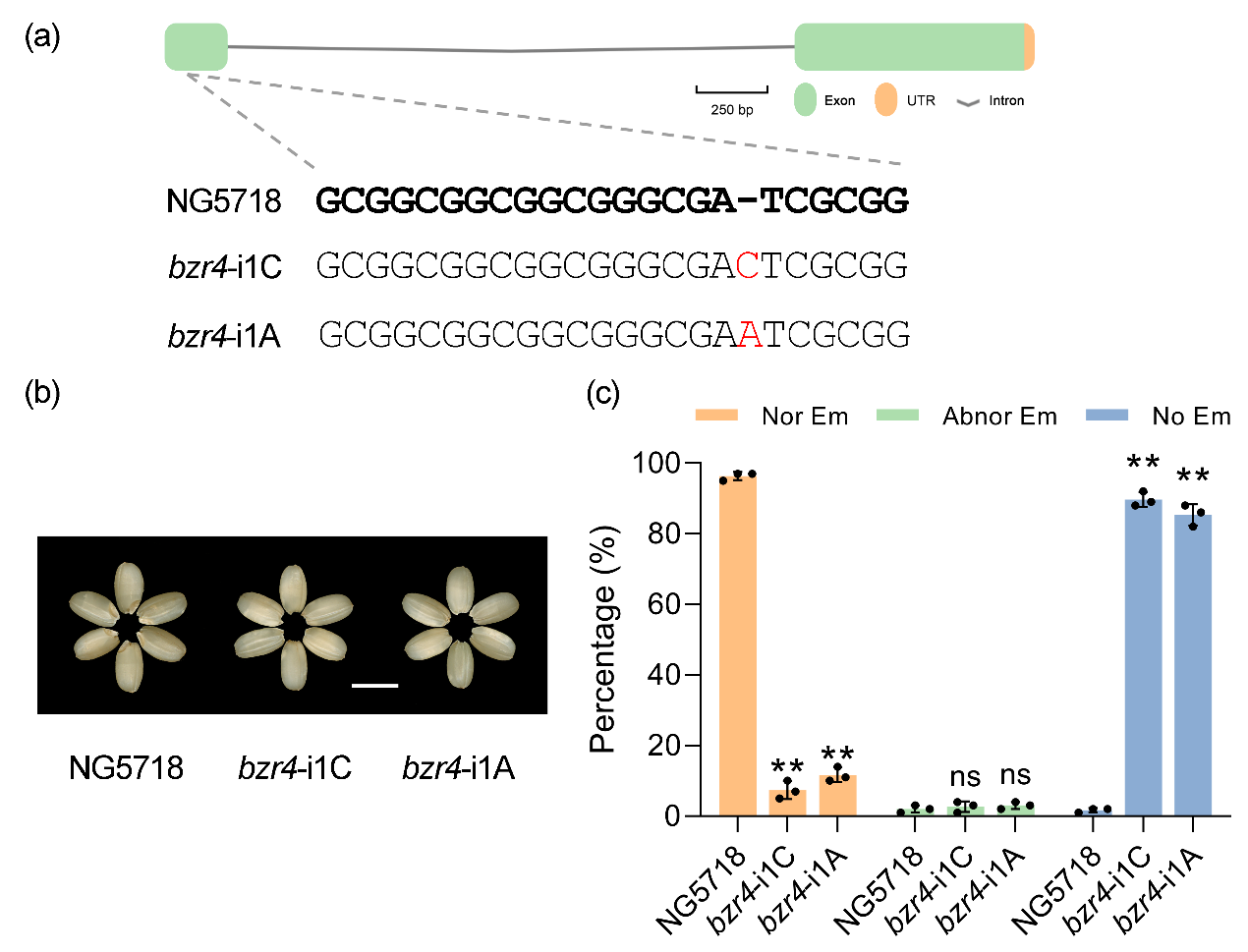
**

**Figure S13** Analysis of *bzr4* mutants in the NG5718 background. (a) Gene editing target in the *BZR4* gene and the corresponding mutation information for the *bzr4* mutants. (b) Embryo phenotypes of *bzr4* mutants. Scale bar, 5 mm. (c) The percentage of different embryo types in the seeds of NG5718 and *bzr4* mutants. All data are means ± SD (n = 3). **P < 0.01; ns, no significance (Student’s *t*-test).

**Supplementary Table**

**Table S1** The primers used in this study

| Primer name | Primer sequence (5'–3') |
| --- | --- |
| BZR4 qRT-F | TCGGGAGCTCCATGACTCG |
| BZR4 qRT-R | AACTTAAACGACGGCCTGCAT |
| GrxC2.2-qRT-F | TAGCCTTCGTTGACCTCTTTATACTG |
| GrxC2.2-qRT-R | CAGCGAACTGCTATCACAAAGACG |
| BBM2-qRT-F | GAAACACCTGCAGCTACGGC |
| BBM2-qRT-R | CTCCAGTTGGCCGAGTAGCC |
| Actin01-F | CCAAGGCCAATCGTGAGAAGA |
| Actin01-R | AATCAGTGAGATCACGCCCAG |
| GrxC2.2-ChIP-PCR-1F | CCTTTTCTTCATGTAATTGTACCCTC |
| GrxC2.2-ChIP-PCR-1R | GCTGAGGGGGCAGAGTTCG |
| GrxC2.2-ChIP-PCR-2F | CAATCTTCTCAATCCACCTCTACTCA |
| GrxC2.2-ChIP-PCR-2R | GCGGTTGCTGGCGGTGGA |
| Actin01(intron)-F | TGGCATCTCTCAGCACATTC |
| Actin01(intron)-R | GGCAAGCAACATTGTAAGCA |
| BZR4-pB42AD-EcoRI-F | gattatgcctctcccgaattcATGATGAACGGAGGAGGGGG |
| BZR4-pB42AD-XhoI-R | agaagtccaaagcttctcgagTTAGCGATCGCCGCGAGT |
| GrxC2.2pro-placzi-1F | cggggatctgtcgacctcgagTTTGTAGAATTTTATTTCCATCTTCTCG |
| GrxC2.2pro-placzi-1R | atacagagcacatgcctcgagGGCAGAGTTCGGAAGGTATGAG |
| GrxC2.2pro-placzi-2F | cggggatctgtcgacctcgagACTCAAACACACCCTAAGAATACGTATA |
| GrxC2.2pro-placzi-2R | atacagagcacatgcctcgagTGGCGGTGGAAGAGCGGT |
| BZR4-pGBD-BamHI-F | ccgtctagaactagtggatccATGATGAACGGAGGAGGGGG |
| BZR4-pGBD-HindIII-R | gtcgacggtatcgataagcttTTAGCGATCGCCGCGAGT |
| BZR4-62-sk-BamHI-F | cgctctagaactagtggatccATGATGAACGGAGGAGGGGG |
| BZR4-62-sk-HindIII-R | gtcgacggtatcgataagcttTTAGCGATCGCCGCGAGT |
| BBM2-62-sk-BamHI-F | cgctctagaactagtggatccATGACCAGGCAGGAGTATATTGC |
| BBM2-62-sk-HindШ-R | gtcgacggtatcgataagcttTCATCCCATGGCGCCATT |
| GrxC2.2-Luc-HindШ-F | gtcgacggtatcgataagcttCTACTCAATTCACAACCTCGCATG |
| GrxC2.2-Luc-BamHI-R | cgctctagaactagtggatccCTCTGCGGTGTGACGTCGC |
| BZR4-BD-EcoRI-F | GGAATTCATGATGAACGGAGGAGGGGGA |
| BZR4-BD-BamHI-R | CGGGATCCTTAGCGATCGCCGCGAGTCA |
| BZR1-AD-EcoRI-F | CGGAATTCATGACGTCCGGGGCGGCGG |
| BZR1-AD-ClaI-R | CCATCGATTCATTTCGCGCCGACGCCGAG |
| GSK2-AD-NdeI-F | GGAATTCCATATGTGGGCACTGGTTCTTTCGG |
| GSK2-AD-SmalI-R | TCCCCCGGGATTTCCTCACGGGTTGGTGT |
| BBM2-AD-EcoRI-F | gccatggaggccagtgaattcATGACCAGGCAGGAGTATATTGC |
| BBM2-AD-BamHI-R | cagctcgagctcgatggatccTCATCCCATGGCGCCATT |
| BZR4-771-KpnI-F | acgggggacgagctcggtaccATGATGAACGGAGGAGGGGG |
| BZR4-771-SalI-R | cgcgtacgagatctggtcgacGCGATCGCCGCGAGTCAT |
| BZR1-772-KpnI-F | tacgcgtcccggggcggtaccATGACGTCCGGGGCGGCG |
| BZR1-772-SalI-R | acgaaagctctgcaggtcgacTCATTTCGCGCCGACGCCGAG |
| GSK2-772-KpnI-F | tacgcgtcccggggcggtaccATGGACCAGCCGGCGCCG |
| GSK2-772-SalI-R | acgaaagctctgcaggtcgacTTAGCTCCCAGTATTGAAGAAGTTGT |
| BBM2-772-KpnI-F | tacgcgtcccggggcggtaccATGACCAGGCAGGAGTATATTGC |
| BBM2-772-SalI-R | acgaaagctctgcaggtcgacTCATCCCATGGCGCCATT |
| BZR4-pKL293-Flag-HindIII-F | atagaataaggagaaaagcttATGATGAACGGAGGAGGGGG |
| BZR4-pKL293-Flag-KpnI-R | gtcatccttgtaatcggtaccGCGATCGCCGCGAGTCAT |
| BZR4-pKL293-Myc-EcoRI-F | tctgaagaagatttggaattcATGATGAACGGAGGAGGGGG |
| BZR4-pKL293-Myc-SalI-R | gtggtggtggtggtggtcgacTTAGCGATCGCCGCGAGT |
| GSK2-pKL293-Myc-EcoRI-F | tctgaagaagatttggaattcATGGACCAGCCGGCGCCG |
| GSK2-pKL293-Myc-Sal1-R | gtggtggtggtggtggtcgacTTAGCTCCCAGTATTGAAGAAGTTGT |
| BZR1-pKL293-Myc-EcoRI-F | tctgaagaagatttggaattcATGACGTCCGGGGCGGCG |
| BZR1-pKL293-Myc-Sal1-R | gtggtggtggtggtggtcgacTCATTTCGCGCCGACGCC |
| BBM2-pKL293-Myc-EcoRI-F | tctgaagaagatttggaattcATGACCAGGCAGGAGTATATTGC |
| BBM2-pKL293-Myc-SalI-R | gtggtggtggtggtggtcgacTCATCCCATGGCGCCATT |
| BBM2-pKL293-Flag-HindШ-F | atagaataaggagaaaagcttATGACCAGGCAGGAGTATATTGC |
| BBM2-pKL293-Flag-KpnI-R | gtcatccttgtaatcggtaccTCCCATGGCGCCATTGAA |
| BZR4-nCFP-Sal1-F | cctactagtggatccgtcgacATGATGAACGGAGGAGGGGG |
| BZR4-nCFP-Sal1-R | agcggtaccctcgaggtcgacTTAGCGATCGCCGCGAGT |
| GSK2-cCFP-Sal1-F | cctactagtggatccgtcgacATGGACCAGCCGGCGCCG |
| GSK2-cCFP-Sal1-R | agcggtaccctcgaggtcgacTTAGCTCCCAGTATTGAAGAAGTTGT |
| BZR1-cCFP-Sal1-F | cctactagtggatccgtcgacATGACGTCCGGGGCGGCG |
| BZR1-cCFP-Sal1-R | agcggtaccctcgaggtcgacTCATTTCGCGCCGACGCC |
| BBM2-cCFP-Sal1-F | cctactagtggatccgtcgacATGACCAGGCAGGAGTATATTGC |
| BBM2-cCFP-Sal1-R | agcggtaccctcgaggtcgacTCATCCCATGGCGCCATT |
| BZR4-pActin-3Flag-SmaI-F | TCCCCCGGGATGATGAACGGAGGAGGGGGAG |
| BZR4-pActin-3Flag-XbaI-R | GCTCTAGAGCGATCGCCGCGAGTCATGG |
| BZR4-seed-F | ggcaGCGGCGGCGGCGGGCGATCG |
| BZR4-seed-R | aaacCGATCGCCCGCCGCCGCCGC |
| GSK2-seed-F | ggcaAGAGACTGTTGCCATTAAGA |
| GSK2-seed-R | aaacTCTTAATGGCAACAGTCTCT |
| BBM2-seed-F | ggcaGGCGCGGCGAGGCGGCTGA |
| BBM2-seed-R | aaacTCAGCCGCCTCGCCGCGCC |
| BZR4-Flag-JCF1 | CTCTCCTCCGCCGGCGTC |
| BZR4-Flag-JCR1 | AAGCAGGGCATGCCTGCA |
| BZR4-Cas-F | ACTGCTTCTCCGGCGTATTT |
| BZR4-Cas-R | AGAGGAAGAGGAAGAGGGACCTTG |
| GSK2-Cas-F | GGGCACTGGTTCTTTCGGTA |
| GSK2-Cas-R | CAAAGGCCATCGCATAGCAAA |
| BBM2-Cas-F | GCGTTTGGGAAACAAAAACCCT |
| BBM2-Cas-R | CGTAGCTGCAGGTGTTTCCA |

**Supplementary Materials and Methods**

**Plant materials**

This study used rice genetic materials related to key genes in BR biosynthesis or signaling pathways, including *GNS4-RNAi* and the wild-type Nipponbare (Nip) (Zhou *et al*., 2017), *d61* and the wild-type Taichung 65 (TC65) (Yamamuro *et al*., 2000), *GSK2-OX*, *dlt* and the wild-type Zhonghua 11 (ZH11) (Tong *et al*., 2012), *BZR1-OX*, *bzr1* and the wild-type Nip (Xiong *et al*., 2022). *BZR4-OX*, *bzr4*, *gsk2*, *bbm2* were generated in the Nip genetic background. In addition, *bzr4* mutants were also generated in the two famous japonica rice cultivars Nangeng 9108 (NG9108) and Nangeng 5718 (NG5718) in Jiangsu Province. All rice materials were cultivated under identical climatic and crop management conditions in Yangzhou, Jiangsu.

**Generation of transgenic rice**

The *BZR4-OX* vector was constructed by cloning the *BZR4* coding sequence (CDS) into the plant binary vector *pCAMBIA1300* under the control of the *OsActin* promoter and fusing it to the 3×FLAG epitope tag. The specific target sites for *BZR4*, *GSK2* and *BBM2* were then designed and cloned into the *pC1300-Cas9* vector (Shen *et al*., 2017; Xie *et al*., 2017) to create the vectors for gene editing. The correct constructs were then transformed into the recipient rice cultivars via Agrobacterium-mediated transformation. All primers used here are listed in Table S1.

**Analysis of embryo size in rice seeds**

After imaging the rice seeds, the cross-sectional areas of both the embryo and the entire seed were quantified using ImageJ software. The embryo-to-seed area ratio was then calculated to provide an indication of the embryo’s relative size. Embryo phenotypes were classified into three groups. The normal embryo (Nor Em) group had an embryo-to-seed ratio equivalent to the corresponding wild type. The abnormal embryo (Abnor Em) group had a significantly lower embryo-to-seed ratio than the wild type. The no embryo (No Em) group consisted of seeds with no observable embryonic structure. Data represent the mean ± SD of three independent biological replicates. Approximately 100 seeds were harvested from each individual plant for analysis in each replicate.

**Pollen fertility evaluation**

This study examined the fertility of rice pollen using the iodine–potassium iodide (I₂–KI) staining method. The detailed experimental procedure is as follows: Anthers exceeding two-thirds of the length of the lemma were considered mature and collected in tubes containing 75 % ethanol (v/v). The spikelets were then removed from the ethanol and 1–2 drops of a 1 % (w/v) I₂-KI solution were added. The anthers were gently crushed with forceps to release the pollen grains. A coverslip was then placed on top. After standing for 2–3 minutes, at least 200 pollen grains were examined at random. Fertile pollen grains are typically round, plump and uniformly dark blue after staining. Sterile pollen grains include those for which no pollen is observed, those that are unstained, those that are morphologically normal but unevenly stained, and those with abnormal morphology.

**Total RNA extraction and RT-qPCR analysis**

Total RNA was extracted from developing rice seeds using an FastPure Universal Plant Total RNA Isolation Kit (Vazyme, China). First-strand cDNA was synthesized using a HiScript III RT SuperMix for qPCR (+gDNA wiper) Kit (Vazyme, China), and RT-qPCR was performed using a ChamQ Universal SYBR qPCR Master Mix (Vazyme, China) on a CFX Connect Real-Time PCR Detection System (Bio-Rad, USA). The *OsActin01* gene was used for normalization, and each experiment included three biological replicates. The primers used are listed in Table S1.

**In situ hybridization analysis**

Digoxigenin-labelled in situ hybridization was performed with minor modifications to the method previously described by Wang *et al*. (2024). The hybridization probe was amplified and then cloned into the pGEM-T vector (Wuhan Corebiolab Co., Ltd., Wuhan, China). The vector was digested with NcoI, purified and then subjected to *in vitro* transcription using a Digoxingenin RNA Labelling Kit (Roche, Basel, Switzerland) according to the manufacturer’s standard protocol. Tissue sample sections were dehydrated using an ethanol series, stained, and then cleared in xylene.

**Histochemical analyses**

Developing seeds at 3, 5, and 15 days after pollination (DAP) were fixed with 3% (w/v) paraformaldehyde, then dehydrated in an ethanol series as previously described (Yi *et al*., 2012). The samples were embedded in paraplast and sectioned to a thickness of 8 μm using a rotary microtome. After staining with 0.05% toluidine blue, the sections were observed using an Olympus BX61 microscope (Olympus, Japan).

**Laser scanning confocal microscope (LSCM)**

Observations using an LSCM were performed as previously described with minor modifications (Huang *et al*., 2017). During peak anthesis, the panicles were gently shaken by hand to promote flowering and facilitate pollination. Developing seeds before pollination and at 1-5 DAP were collected and fixed in FAA (50% ethanol, acetic acid and formaldehyde at a volume ratio of 89:5:6) for at least 24 hours. These were then rehydrated using a graded ethanol series and distilled water. Spikelets collected one day before anthesis were used for photography and morphological observation of the glumes, anthers, and ovaries. These samples were then mordanted with aluminum potassium sulfate (2%) for 20 minutes, stained with eosin B (10 mg/L) for 16 hours, washed with distilled water, dehydrated using an ethanol series, and infiltrated with a solution of ethanol and methyl salicylate (1:1, v/v) overnight. The samples were then incubated in methyl salicylate. The infiltrated caryopses were observed using an LSM 800 confocal microscope (Carl Zeiss, Oberkochen, Germany) with excitation/emission wavelengths of 543 nm.

**Western blotting analysis**

Rice leaf samples were weighed and ground into a powder in liquid nitrogen, and the total protein was extracted and denatured at 99°C for 10 minutes. This was then separated by SDS-PAGE and then transferred to a PVDF membrane. An antibody that specifically recognizes the Flag tag protein was used.

**Yeast one-hybrid assay**

The CDS of the *BZR4* gene was cloned into the *pB42AD* vector. A 1795 bp promoter sequence of the *GrxC2.2* gene was cloned into the *pLacZi* vector. The two constructs were co-transformed into the EGY48 yeast strain. The interaction between the BZR4 protein and the *GrxC2.2* promoter was confirmed by the presence of blue colonies on the screening medium.

**Yeast two-hybrid assay**

The CDS of the *BZR4* gene was cloned into the *pGBKT7* vector. The generated *BZR4-pGBKT7* construct was used as the bait for yeast two-hybrid screening. The CDS of the *BZR1*, *GSK2* and *BBM2* genes were cloned into the *pGADT7* vector. The resulting *BZR1-*, *GSK2-* and *BBM2-pGADT7* constructs were co-transformed into the AH109 yeast strain alongside the *BZR4-pGBKT7* construct in order to verify direct protein-protein interactions with BZR4.

**Pull-down experiment**

The CDS of *BZR4*, *BZR1*, *GSK2* and *BBM2* were cloned into the *pKL293-MFH* vector to create the following plasmids: *BZR4-pKL293-Flag*, *BZR1-pKL293-Myc*, *GSK2-pKL293-Myc* and *BBM2-pKL293-Myc*. The target proteins were then expressed using the TnT® SP6 high-yield wheat germ protein expression system (Promega, USA). The GFP-Flag fusion protein, which was generated by expressing the *pKL293-MFH* empty vector, was used as the negative control. The pull-down experiment was performed according to the methods described in a previous publication (Li *et al*., 2016), and the primer sequences are listed in Table S1.

**Split-luciferase complementation assay (SLCA)**

The vectors *JW771* and *JW772* were used for the SLCA. The CDS of *BZR4* was cloned into vector *JW771*, and the CDS of *BZR1*, *GSK2* and *BBM2* were cloned into vector *JW772*. The generated constructs were transformed into *Nicotiana benthamiana* leaves via Agrobacterium-mediated transformation. The detailed SLCA protocol can be found in the aforementioned publication (Wang *et al*., 2024).

**Bimolecular fluorescence complementation (BiFC) assay**

The CDS of *BZR4* was cloned into the *cCFP* vector, and the CDS of *BZR1*, *GSK2* and *BBM2* were cloned into the *nCFP* vector. The *BZR4*-*cCFP* and the respective *BZR1*, *GSK2-* and *BBM2-nCFP* plasmids were co-transformed into rice protoplast cells and incubated overnight at 25 °C in the dark. The fluorescence signal was finally observed using a confocal laser scanning microscope (LSM710, Carl Zeiss, Germany). Primer sequences are listed in Table S1.

**Chromatin immunoprecipitation (ChIP)-qPCR assay**

The ChIP assay was performed as previously described (Xiong *et al*., 2022). Briefly, young panicles of *BZR4-OX* transgenic rice were used for the analysis. An anti-Flag monoclonal antibody conjugated to immunomagnetic beads (TransGen) was used to immunoprecipitate the protein-DNA complex. The precipitated DNA fragments were analyzed by RT-qPCR to evaluate the enrichment effect. The expression of a region of the *OsActin01* intron was used for normalization, and the *GrxC2.2* gene was used as the positive control. Analysis of each DNA region was repeated three times. The primers used are shown in Table S1.

**Subcellular localization analysis**

The CDS of *BZR4* was ligated into the *pCAMBIA2300-35S-GFP* vector to create the *BZR4-GFP* construct. Agrobacteria (GV3101) containing the *BZR4-GFP* construct were then infiltrated into the tobacco leaves. The resulting fluorescence signal was observed using an LSM710 confocal laser scanning microscope (Carl Zeiss, Germany). Primer sequences are listed in Table S1.

**Dual-luciferase reporter assay**

The *GrxC2.2* promoter was cloned into the *pGreenII 0800-LUC* vector to create the *GrxC2.2pro-LUC* reporter construct. The *BZR4* CDS and the *BBM2* CDS were cloned into the *pGreenII BD* and *pGreenII 62-sk* vectors, respectively, to generate the *BZR4-pGreenII BD*, *BZR4-pGreenII 62-sk* and *BBM2-pGreenII 62-sk* plasmids. Plasmid combinations were co-transformed into rice protoplasts according to the experimental design. The transformed cells were then incubated in the dark at 28 °C for 12 hours. Transcriptional activity was subsequently measured using a dual luciferase assay kit (Vazyme Biotech, Jiangsu, China).

**Statistical analysis**

In this study, all data are presented as the mean ± SD. For experiments involving single pairwise comparisons, a Student’s *t*-test was used to determine the level of significance (*P < 0.05, **P < 0.01). For experiments involving multiple comparisons, Duncan’s multiple range test was used at P < 0.05, with different lowercase letters indicating statistical significance.

**Supplementary References**

Huang, X., Peng, X., Sun, M. X. (2017) *OsGCD1* is essential for rice fertility and required for embryo dorsal-ventral pattern formation and endosperm development. *New Phytol*. **215**, 1039–1058.

Li, Q., He, J. (2016) BZR1 Interacts with HY5 to mediate brassinosteroid- and light-regulated cotyledon opening in *Arabidopsis* in darkness. *Mol Plant*, **9**, 113-125.

Shen, L., Hua, Y., Fu, Y., Li, J., Liu, Q., Jiao, X., Xin, G. *et al*. (2017) Rapid generation of genetic diversity by multiplex CRISPR/Cas9 genome editing in rice. *Sci China Life Sci*. **60**, 506–515.

Tong, H., Liu, L., Jin, Y., Du, L., Yin, Y., Qian, Q., Zhu, L. *et al*. (2012) DWARF AND LOW-TILLERING acts as a direct downstream target of a GSK3/SHAGGY-like kinase to mediate brassinosteroid responses in rice. *Plant Cell*, **24**, 2562-77.

Wang, J., Wang, J., Huang, L., Kan, L., Wang, C., Xiong, M., Zhou, P. *et al*. (2024) ABA-mediated regulation of rice grain quality and seed dormancy via the NF-YB1-SLRL2-bHLH144 Module. *Nat. Commun*. **15**, 4493.

Xie, X., Ma, X., Zhu, Q., Zeng, D., Li, G., Liu, Y. (2017) CRISPR-GE: a convenient software toolkit for CRISPR-based genome editing. *Mol Plant*, **10**, 1246–1249.

Xiong, M., Yu, J., Wang, J., Gao, Q., Huang, L., Chen, C., Zhang, C. *et al*. (2022) Brassinosteroids regulate rice seed germination through the BZR1-*RAmy3D* transcriptional module. *Plant Physiol*. **189**, 402-418.

Yamamuro, C., Ihara, Y., Wu, X., Noguchi, T., Fujioka, S., Takatsuto, S., Ashikari, M.*et al*. (2000) Loss of function of a rice brassinosteroid insensitive1 homolog prevents internode elongation and bending of the lamina joint. *Plant Cell*, **12**, 1591-606.

Yi, J., Kim, S., Lee, D., Moon, S., Lee, Y., Jung, K., Hwang, I. *et al*. (2012) The rice gene *DEFECTIVE TAPETUM AND MEIOCYTES 1 (DTM1)* is required for early tapetum development and meiosis. *Plant J*. **70**, 256–270.

Zhou, Y., Tao, Y., Zhu, J., Miao, J., Liu, J., Liu, Y., Yi, C. *et al*. (2017) *GNS4*, a novel allele of *DWARF11*, regulates grain number and grain size in a high-yield rice variety. *Rice*, **10**, 34.
